# Clinical Recollections

**Published:** 1896-03-14

**Authors:** Benjamin Ward Richardson


### Clinical Recollections.

By Sir Benjamin Ward Richardson, M.D., F.R.S.

### ALCOHOL.

In my last paper I dwelt on alcohol when administered in its medicinal form, and ventured to show that it was not merely an idea on which it rested, but that it had certain defined and distinctive effects which may be called medicinal in their character, if we take that view of the case. I showed that it obliterated pain under certain circumstances, being a direct anæsthetic, or soother of pain. I said it had been suggested that it had the power of stopping waste of tissues, and that it possessed unquestionable properties which led it to be considered a stimulant, that is to say, a substance which excited into quicker action—while it was in action—both the heart and arterial system. I mentioned incidentally also that many are inclined to look upon alcohol as a food, and as something that will repair the tissues equally well as any other tissue-supplying material.

The statements made seem to me to suggest that those who use alcohol have some lines of argument in their favour, although I do not myself admit them as sound. But there is another point to be considered, which is that usage has led millions of people to consider that alcohol is of service, not simply as a medicinal stimulant in the treatment of disease, but as an agent which may be necessary for the common wants of the body. We constantly hear it said in daily life that this or that person is "running down," and should be, therefore, supplied with wine or other alcoholic beverages. Again, we hear it said that such and such a person is losing flesh, or is becoming low from the production and elimination of a secretion, and, therefore, should be supported by alcohol. In the case of women who are suckling children it is an every-day argument that the drain produced can only be met by the administration of a beverage containing alcohol like stout or porter. When a person has to do a great deal of physical work, by which he is fatigued, it is a common practice to indicate that so soon as the work begins to tell on the body an alcoholic should be given in order to sustain the fatigue; or, if the labour or strain be mental instead of physical, and produces fatigue—for mental work does, indeed, produce physical exhaustion—the argument is re-applied, and it is suggested that alcohol is being called for in order to meet the demand that is imposed. Most striking of all is it that when the body from any cause is reduced by disease from excessive elimination, or more frequently from failure of nervous power, it is declared urgently that some kind of alcoholic drink is necessary—especially at meals—in order that the structure and functions of the body may be maintained. One of the worst features in our profession lies, not in the mere administration of alcohol, but in the circumstance that there is no rule of any special order amongst medical men that leads to the selection of any particular fluid. In my consulting-room in one morning I have heard people say that doctors have recommended for them as many representatives of alcoholic drinks as they themselves numbered. One doctor is all for ales and stouts, and very rarely goes out of his way to consider whether they are the best of the series in hand; another goes in entirely for whisky; says there is nothing like sound, good whisky to take either with or without solid food, giving no particular direction as to what quantities of water shall be taken with it, or in what particular quantities it shall be taken; a third recommends wine, dealing sometimes with a particular wine, but usually employing the term in a general sense, and making little, if any, distinction between port and sherry, burgundy and claret, champagne or hock; a fourth will pin his faith on brandy, which, diluted with water, will, he thinks, answer every purpose, and a few will speak favourably of gin, particularly in examples where some diseases of the kidney are under observation. All these variations are exceedingly unfortunate, because the people prescribed for almost invariably accept them as indefinite, and as meaning that after all they are simply prescribed alcohol without any clear understanding as to what is the precise nature of the substances they have to imbibe. I tried many years ago to meet these objections, and to advance that, as a remedy, alcohol itself should be supplied, not in any form of mixture as a beverage, but as alcohol itself diluted with water. I thought that anyone brought up after the usual method of life might take a given quantity of alcohol, say, half an ounce to an ounce and a-half a-day, properly diluted, with good effects, and with a certainty that could not otherwise be obtained. I soon found, however, that the practice was a dangerous one, for if it be once admitted that alcohol in some form is necessary, patients will make their own choice and take that form they like best. A most distinguished man, a high ecclesiastic, explained to me—as did also his medical man—that he could not live, to say nothing about work, unless I would prescribe alcohol for him in this fashion. It appeared just reasonable, so the doctor and I together did the thing. We wrote a prescription in which alcohol was duly prescribed in combination with water, and the patient, who was himself most anxious to sustain abstinence, commenced to take it; but a clever wine-seller acquiescing in this matter requested that he might be shown the prescription, and having got it into his hands, explained that he possessed a wine which contained precisely the same measure, or weight, of alcohol, and that, therefore, his wine might just as well be taken as our physic. Also in a drawing-room another ecclesiastic, in a kind of boast or joke, said that I prescribed wine, although on being driven to produce the prescription, he explained that wine was not in my prescription, but that he indulged in a wine containing just the same amount of alcohol; and if a lady present had not taken my part very firmly, and if the patient had not apologised afterwards for what he had said, I should have stood at a low mark in regard to the prescription. I do not, therefore, recommend this plan except on very rare occasions, and I do not think that for three years I have resorted to it, although many medical men have written to ask me what the proportions are in which I prescribe pure alcohol in combination with water.

